# Cardiac rehabilitation barriers by rurality and socioeconomic status: a cross-sectional study

**DOI:** 10.1186/1475-9276-12-72

**Published:** 2013-08-28

**Authors:** Shamila Shanmugasegaram, Paul Oh, Robert D Reid, Treva McCumber, Sherry L Grace

**Affiliations:** 1 York University, Toronto, Canada; 2 University Health Network - Toronto Rehabilitation Institute, Toronto, Canada; 3 University of Ottawa Heart Institute, Ottawa, Canada; 4 Mackenzie Health, Toronto, Canada; 5 York University and University Health Network, Toronto, Canada

**Keywords:** Cardiac rehabilitation, Rural, Socioeconomic status

## Abstract

**Introduction:**

Despite greater need, rural inhabitants and individuals of low socioeconomic status (SES) are less likely to undertake cardiac rehabilitation (CR). This study examined barriers to enrollment and participation in CR among these under-represented groups.

**Method:**

Cardiac inpatients from 11 hospitals across Ontario were approached to participate in a larger study. Rurality was assessed by asking participants whether they lived within a 30-minute drive-time from the nearest hospital, with those >30 minutes considered “rural.” Participants completed a sociodemographic survey, which included the MacArthur Scale of Subjective Social Status. One year later, they were mailed a survey which assessed CR utilization and included the Cardiac Rehabilitation Barriers Scale. In this cross-sectional study, CR utilization and barriers were compared by rurality and SES.

**Results:**

Of the 1809 (80.4%) retained, there were 215 (11.9%) rural participants, and the mean subjective SES was 6.37 ± 1.76. The mean CRBS score was 2.03 ± 0.73. Rural inhabitants reported attending significantly fewer CR sessions (p < .05), and greater CR barriers overall compared to urban inhabitants (p < .01). Patients of lower subjective SES were significantly less likely to be referred, enroll, and participate in CR, and reported significantly greater barriers to CR compared to their high SES counterparts (p < .01). Prominent barriers for both groups included distance, cost, and transportation problems. These relationships sustained adjustment, and a significant relationship between having undergone coronary artery bypass graft surgery and lower barriers was also identified.

**Conclusions:**

The results confirm that rural inhabitants and patients of low SES experience greater barriers to CR utilization when compared to their urban, high SES counterparts. It is time to implement known strategies to overcome these barriers, to achieve equitable and greater use of CR.

## Introduction

Heart disease is one of the leading causes of mortality and morbidity worldwide [1,2]. Patients with heart disease benefit significantly from participation in comprehensive cardiac rehabilitation (CR) programs, and many clinical practice guidelines promote CR as a standard part of the continuum of care [3]. CR is an outpatient approach to the secondary prevention of heart disease, and it is composed of structured exercise, comprehensive education, and counseling. CR reduces mortality by 25% as well as hospital readmissions, interventional procedures and cardiac risk factors, and improves well-being among both men and women [4,5]. Despite the well-established benefits of CR, it is significantly under-utilized [6]. In particular, the rates of CR utilization among rural inhabitants and patients of low socioeconomic status (SES) are low [7]. Given that these under-represented populations are at greater risk of suffering from heart disease and its long-term consequences, [7] this represents another disturbing example of the treatment-risk paradox [8].

### Rurality

The term “rural” refers to the population living in towns and municipalities outside the commuting zone of larger urban centers (i.e., with a population of 10,000 or more) [9]. Evidence suggests that rural inhabitants generally have a constellation of risk factors that put them at greater need for CR [7]. For instance, rural patients are more likely to be smokers [10] and less physically active [10], compared to their urban counterparts.

Despite their greater need for CR [7], research suggests that rural inhabitants are less likely to undertake CR compared to their urban counterparts [11]. Suaya et al. [12] used patients’ residence zip codes to determine distance to the closest CR site, and found that patients in the furthest quintile group, with a mean distance of 31.8 miles, were 71% less likely to utilize CR. Research suggests that barriers that are geographic in nature such as CR site location and distribution, distance, transportation access, parking costs, and patient driving status are significant barriers for patients from rural areas [11-15]. In addition, other barriers that may influence CR utilization among rural patients include quality of roads and harsh weather conditions [16,17].

### Socioeconomic status

SES is defined as a hierarchical continuum on the basis of prestige, lifestyle, attitudes, and values, which can define a person’s position in society [18]. There is literature to suggest that low SES is associated with greater morbidity and mortality among coronary heart disease patients [19-22]. Alter et al. [23] found that the prevalence of diabetes, hypertension, smoking, and pre-existing heart disease were higher among poorer, less-educated patients with acute myocardial infarction, as were the total number of cardiovascular risk factors. This reiterates the need for CR in low SES patients.

Suaya et al. [12] found that patients living in zip codes with the highest median household income were 23% more likely to participate in CR than those living in zip codes with the lowest median income. Research suggests that barriers to CR participation among patients of low SES include fewer health benefits such as paid time off from work for preventive health programs, program expense and insurance coverage, and transportation issues [7].

Although the nature of barriers that rural inhabitants and patients of low SES experience have been described previously [7], to date, there is lack of research that compares barriers among these vulnerable groups with their respective counterparts using a psychometrically-validated and comprehensive CR barriers scale. Moreover, much of the research in this area has stemmed from the United States where cost of CR is a formidable barrier for low SES patients. Ultimately, these barriers may be amenable to modification or intervention [24,25], thus potentially increasing CR utilization and facilitating optimal patient recovery and outcomes among these under-represented groups. Accordingly, the objectives of the current study were to compare: [1] CR utilization, and [2] barriers to CR utilization among rural versus urban patients, as well as patients of low versus high SES. It was hypothesized that rural patients and those of low SES would report greater barriers to CR utilization.

## Method

### Design and procedure

This is a secondary analysis of a larger study called Cardiac Rehabilitation care Continuity through Automatic Referral Evaluation (CRCARE) [26], comparing CR enrollment following different means of referral with quota sampling of under-represented groups. Ethics approval was granted from all participating institutions. CR services are reimbursed through provincial health care (although patients pay for transportation and/or parking at each visit) in the province where this study was conducted.

Cardiac inpatients from 11 hospitals across Ontario were approached to participate in the larger study. Recruiters went to the applicable inpatient unit each business day to ask the nurses whether they were providing care to patients who met study diagnosis criteria. Potentially eligible patients were first approached by someone in their circle of care, to request verbal consent to speak to a recruiter about the study. Upon obtaining written informed consent, clinical data were extracted from medical charts, and a self-report survey with a pre-postage stamped envelope were provided to patients. Patients were asked to complete the survey either during their hospitalization or at home. Among other variables, this survey assessed sociodemographic characteristics. One year later, participants were mailed a follow-up survey assessing CR participation and barriers. The cross-sectional analyses herein were based on this latter cohort of retained participants.

### Participants

The inclusion criteria for the larger study were: confirmed acute coronary syndrome diagnosis, patients who had undergone percutaneous coronary intervention, coronary artery bypass graft surgery, or valve repair/replacement or had heart failure. These diagnostic criteria were chosen based on Canadian CR clinical practice guideline recommendations for indicated patients [3]. The exclusion criteria for the larger study were: participation in CR within the past two years, and significant orthopedic, neuromuscular, visual, cognitive and/or any serious mental illness which would preclude CR participation. A total of 2635 stable cardiac inpatients were recruited.

### Measures

Rurality was operationalized in relation to drive time to hospital. A 30-minute drive time standard for “accessible” hospital care was originally identified by Bosanac, Parkinson, and Hall [27]. Research has shown that as time of travel increases over 30 minutes, patients are less likely to utilize health care services[28,29] including CR [30]. The Cardiac Care Network of Ontario Consensus Panel recommended this drive time threshold to define accessible CR. Accordingly, in the current study, patients were asked at the time of recruitment whether they lived within a 30-minute drive of a hospital, and were coded as rural if they responded “no.”

The MacArthur Scale of Subjective Social Status was administered in the in-hospital survey [31]. SES can be assessed using objective and/or subjective indicators. Objective indicators of SES refer to income, education, and work status, and these were assessed in the in-hospital survey. On the MacArthur Scale of Subjective Social Status, participants were asked to demarcate their socioeconomic status on a 10-rung ladder compared to others in Canada. Scale scores ranged from 1 to 10, with higher scores indicating greater subjective socioeconomic status. A median split was computed, to categorize participants as high versus low subjective SES.

Self-reported sociodemographic variables measured in the survey administered in-hospital also included patients’ marital status and ethnocultural background (response options were based on Statistics Canada). Sociodemographic data obtained from the medical chart included date of birth and sex. Clinical variables obtained from the chart included body mass index (kg/m^2^), diabetes mellitus, hypertension, dyslipidemia, smoking status, reason for cardiac admission, and comorbidities.

The one-year follow-up survey assessed self-reported CR utilization, through forced-choice response options for referral (yes/no), enrollment (yes/no), and participation (yes/no). Patients were also asked to estimate the percentage of prescribed CR sessions attended (0-100%).

The Cardiac Rehabilitation Barriers Scale (CRBS) assesses patients’ perceptions of the degree to which patient, provider, and health system-level barriers affect their CR enrollment and participation [32]. Regardless of CR referral or enrollment, participants were asked to rate their level of agreement with the 21 statements. Items were rated on a 5-point Likert-type scale that ranged from 1 = *strongly disagree* to 5 = *strongly agree*. A mean score was computed, with higher scores indicating greater barriers to patient enrollment or participation in a CR program. The CRBS is demonstrated to be a valid and reliable measure [32].

### Statistical analyses

SPSS Version 20.0 [33] was used to analyze the data. First, *t*-tests and chi-square analyses were performed to assess differences in sociodemographic and in-hospital clinical characteristics between rural versus urban patients and those of low versus high SES. Second, chi-square tests were performed to test differences in CR referral, enrollment, and participation among these subgroups. *T*-tests were used to assess differences in percentage of prescribed CR sessions attended in each of the subgroups.

To test the objective of the study, a descriptive examination of CR barriers was first performed by subgroup. T-tests were then used to assess whether there were significant differences in total barriers, and for each barrier item by rurality and SES. A Bonferroni correction was applied to control against inflated error due to multiple comparisons for the latter tests, such that a p-value < .002 (.05/21) was considered statistically significant.

Next, we used Pearson’s r to assess the relationships between sociodemographic and clinical variables and CRBS. Finally, a General Linear Model was used to assess whether rurality and SES (independent variables) were still related to total CRBS score (dependent variable), after adjusting for the sociodemographic and clinical variables that were significantly related to CRBS identified through the bivariate analyses outlined above and in the literature.

## Results

### Respondent characteristics

The sample for this study comprised 1809 participants (80.4% retention rate) who completed the one-year follow-up survey. Two patients did not provide information regarding their rural/urban status, whereas 178 participants did not complete the MacArthur Scale of Subjective Social Status. There were some significant differences in the characteristics of participants retained versus those lost to follow-up that are reported elsewhere [26]. Of these participants, 939 (51.9%) participated in CR, at one of 61 sites. The mean CRBS score was 2.03 ± 0.73.

Table 1 displays the sociodemographic and clinical characteristics of the participants by rural versus urban residence, and low versus high SES. Rural patients were significantly less often male and had lower rates of coronary artery bypass graft surgery and/or percutaneous coronary intervention compared to urban patients. Patients of low SES were significantly less often male and married, and more often earned lower income annually compared to patients of high SES. In addition, they were more often smokers, and had diabetes and comorbidities.

**Table 1:** Sociodemographic and clinical characteristics of participants by rurality and socioeconomic status

**Mean ± SD / n (%)**	**Rural (n = 215)**	**Urban (n = 1592)**	**Low SES (n = 726)**	**High SES (n = 905)**	**Total (N = 1807)**
**Sociodemographic characteristics**					
Age	65.0 ± 10.5	65.4 ± 10.4	64.9 ± 10.7	65.5 ± 10.1	65.4 ± 10.4
Sex (% men)	147 (68.4)	1208 (75.9)*	524 (72.2)	719 (79.4)**	1357 (75.0)
Ethnicity (% white)	173 (85.2)	1271 (83.2)	580 (82.4)	762 (85.3)	1446 (83.4)
Marital status (% married)	169 (79.0)	1223 (77.6)	517 (72.2)	739 (82.3)***	1392 (77.8)
Education (% ≥ High school)	142 (69.3)	1170 (75.6)	467 (66.1)	731 (83.7)***	1312 (74.8)
Work status (% Retired)	110 (54.2)	793 (51.7)	371 (52.0)	465 (52.1)	905 (52.0)
Annual family income (% ≥ $50,000CAD)	72 (44.4)	656 (50.7)	187 (30.9)	511 (66.5)***	730 (50.0)
**Clinical characteristics**					
Body mass index	29.5 ± 5.99	29.0 ± 5.35	29.6 ± 6.15*	28.8 ± 4.90	29.0 ± 5.40
Coronary artery bypass graft surgery	72 (33.5)	670 (42.4)*	286 (39.7)	385 (42.8)	743 (41.3)
Myocardial infarction	59 (27.4)	442 (28.0)	210 (29.2)	237 (26.3)	502 (28.0)
Percutaneous coronary intervention	59 (27.4)	543 (34.3)*	233 (32.3)	318 (35.3)	602 (33.5)
Heart failure	24 (11.2)	170 (10.7)	88 (12.2)	84 (9.3)	194 (10.8)
Valve (Repair)	5 (31.2)	36 (28.1)	19 (31.1)	13 (20.0)	41 (28.5)
Diabetes mellitus	68 (33.8)	449 (31.1)	235 (35.6)**	234 (28.3)	517 (31.5)
Family history of cardiovascular disease	105 (67.3)	749 (64.4)	344 (65.3)	428 (64.5)	854 (64.7)
Hypertension	139 (70.2)	1100 (74.6)	500 (74.4)	615 (73.4)	1239 (74.1)
Hypercholesterolemia	145 (81.5)	1139 (81.9)	513 (81.6)	657 (82.4)	1284 (81.9)
Smoker	13 (6.2)	98 (6.4)	55 (7.6)*	44 (4.9)	111 (6.4)
Comorbidities	132 (67.0)	982 (68.0)	469 (71.2)*	539 (65.6)	1114 (67.8)

*p < .05; **p < .01; ***p < .001.

*SD* standard deviation, *SES* socioeconomic status, *CAD*, Canadian dollar.

### Rurality

There were 215 (11.9%) participants considered rural. As shown in Table 2, there were no significant differences between rural and urban patients in terms of referral, enrollment, or participation in CR. However, rural inhabitants reported attending a significantly lower percentage of CR sessions compared to urban inhabitants.

**Table 2:** Self-reported cardiac rehabilitation referral, enrollment, participation and barriers by rurality and socioeconomic status

	**Rural (n = 215; 11.9%)**	**Urban (n = 1592; 88.0%)**	**Low SES (n = 726; 40.1%)**	**High SES (n = 905; 50.0%)**
CR referral	131 (63.0)	1024 (65.1)	440 (61.4)	606 (68.1)**
CR enrollment	110 (53.1)	867 (56.7)	360 (51.6)	525 (60.2)**
CR participation	100 (47.6)	838 (54.0)	355 (50.3)	497 (56.1)*
% CR sessions completed†	76.2 ± 31.5	83.6 ± 26.6*	81.1 ± 29.5	84.8 ± 24.5
CRBS Total score†	2.24 ± 0.73**	2.00 ± 0.73	2.15 ± 0.76***	1.94 ± 0.68

*p < .05, **p < .01; ***p < .001.

†mean ± standard deviation.

*SES* socioeconomic status, *CR* cardiac rehabilitation, *CRBS* cardiac rehabilitation barriers scale.

As shown in Table 2, rural participants reported significantly greater total CR barriers than their urban counterparts (t = 3.51, p < .001). Rural participants perceived some of their greatest barriers to CR as: already exercising at home or in one’s community, distance, and cost. As shown in Table 3, rural participants rated the following barriers significantly greater than urbanites: distance, cost, transportation problems, severe weather, and family responsibilities.

**Table 3:** Mean cardiac rehabilitation barrier scores (± standard deviation) by rurality and socioeconomic status

**Barriers**	**Rural (n = 215; 11.9%)**	**Urban (n = 1592; 88.0%)**	**Low SES (n = 726; 40.1%)**	**High SES (n = 905; 50.0%)**	**Total (N = 1809)**
Travel	2.38 ± 1.19	2.29 ± 1.31	2.19 ± 1.18	2.42 ± 1.39	2.31 ± 1.30
I already exercise at home or in my community	3.01 ± 1.37	2.84 ± 1.43	2.91 ± 1.38	2.85 ± 1.45	2.86 ± 1.42
Work responsibilities	2.47 ± 1.35	2.19 ± 1.27	2.27 ± 1.26	2.20 ± 1.31	2.22 ± 1.28
Time constraints	2.41 ± 1.27	2.10 ± 1.21	2.15 ± 1.19	2.15 ± 1.25	2.14 ± 1.22
Severe weather	2.55 ± 1.32*	2.16 ± 1.29	2.38 ± 1.34*	2.05 ± 1.25	2.21 ± 1.30
Other health problems prevent me from going	2.24 ± 1.27	2.11 ± 1.26	2.23 ± 1.29	2.01 ± 1.20	2.13 ± 1.27
I find exercise tiring or painful	2.47 ± 1.34	2.17 ± 1.23	2.33 ± 1.27*	2.08 ± 1.20	2.20 ± 1.25
Distance	2.91 ± 1.49*	2.21 ± 1.38	2.50 ± 1.46*	2.13 ± 1.35	2.30 ± 1.42
Family responsibilities	2.31 ± 1.13*	1.94 ± 1.15	2.10 ± 1.17	1.89 ± 1.13	1.98 ± 1.15
Cost	2.63 ± 1.45*	2.11 ± 1.30	2.38 ± 1.37*	1.98 ± 1.25	2.18 ± 1.33
I don’t have the energy	2.38 ± 1.31	2.07 ± 1.18	2.24 ± 1.24*	1.97 ± 1.13	2.11 ± 1.20
Transportation problems	2.48 ± 1.41*	1.96 ± 1.67	2.24 ± 1.26*	1.82 ± 1.12	2.02 ± 1.21
I prefer to take care of my health alone	2.37 ± 1.24	2.11 ± 1.21	2.22 ± 1.19	2.08 ± 1.22	2.14 ± 1.21
It took too long to get referred and into the program	2.21 ± 1.20	1.88 ± 1.07	2.08 ± 1.14*	1.77 ± 0.99	1.92 ± 1.09
I can manage on my own	2.15 ± 1.08	2.03 ± 1.13	2.11 ± 1.12	1.99 ± 1.10	2.05 ± 1.12
I don’t need CR	2.22 ± 1.26	2.17 ± 1.27	2.23 ± 1.27	2.12 ± 1.25	2.18 ± 1.27
Many people with heart problems don’t go to CR and they are fine	2.14 ± 1.10	1.89 ± 1.02	2.06 ± 1.07*	1.82 ± 0.99	1.92 ± 1.03
My doctor didn’t feel it was necessary	2.07 ± 1.08	2.03 ± 1.18	2.13 ± 1.18	1.93 ± 1.13	2.04 ± 1.17
I am too old	1.85 ± 0.96	1.72 ± 0.94	1.86 ± 0.98*	1.63 ± 0.89	1.74 ± 0.95
I think I was referred but the rehab program didn’t contact me	2.01 ± 1.14	1.80 ± 1.05	1.97 ± 1.11*	1.70 ± 1.00	1.82 ± 1.06
I didn’t know about CR	2.36 ± 1.40	2.13 ± 1.40	2.33 ± 1.44*	1.99 ± 1.34	2.16 ± 1.40

*p < .002 for rural versus urban or high versus low SES comparison.

*SES* socioeconomic status, *CR* cardiac rehabilitation.

### Socioeconomic status

The mean subjective SES score was 6.37 ± 1.76 (median = 6.50). As shown in Table 2, patients of low subjective SES reported significantly lower referral, enrollment, and participation in CR than those of high SES. There was no significant difference between patients of low versus high SES in terms of percentage of CR sessions attended.

As shown in Table 2, participants who rated themselves below the median on the subjective SES ladder reported significantly greater total CR barriers compared to those above (t = 4.47, p < .001). Patients of low SES perceived some of their greatest barriers to CR as: already exercising at home or in one’s community, distance, severe weather, and cost. As shown in Table 3, barriers that were significantly greater for patients of low SES were severe weather, distance, cost, transportation problems, I find exercise tiring or painful, I am too old, I don’t have the energy, it took too long to get referred and into the program, I think I was referred but the rehab program didn’t contact me, many people with heart problems don’t go to CR, and they are fine, and I didn’t know about CR when compared to patients of high SES.

Finally, a univariate analysis of variance with multiple predictors (factors) was run to ascertain whether the association between rurality and SES with CR barriers remained. The model was adjusted for sociodemographic and clinical characteristics shown to be associated with the CRBS at the bivariate level (Table 4) and in the literature [12]. Income and education were not included in the model as they would be confounded with subjective SES. As shown in Table 5, the interaction between rurality and subjective SES was not significant. The significant difference in CR barriers among rural versus urban [F(1, 846) = 4.61, p < .05] and low SES versus high SES [F(1, 846) = 13.45, p < .001] patients sustained adjustment.

**Table 4:** Correlates of mean total cardiac rehabilitation barriers scale

**Variable**	**Mean total CRBS (r)**
Age	.06
Sex	.11**
Ethnicity	.02
Marital status	.09**
Education	.14***
Work status	-.02
Annual family income	.21***
Body mass index	.02
Coronary artery bypass graft surgery	.15***
Myocardial infarction	-.03
Percutaneous coronary intervention	-.08*
Heart failure	-.04
Valve (Repair)	-.07
Diabetes mellitus	-.07*
Family history of cardiovascular disease	.01
Hypertension	-.01
Hypercholesterolemia	-.01
Smoker	-.03
Comorbidities	-.05

*p < .05; **p < .01; ***p < .001.

*CRBS* cardiac rehabilitation barriers scale.

**Table 5:** General linear model assessing association with mean total cardiac rehabilitation barriers (N = 854)

**Variable**	**F**	**df**	**p**
Sex	4.52	1	<.05
Marital status	2.71	1	.10
CABG	17.11	1	<.001
Comorbidities	1.84	1	.18
Rural	4.61	1	<.05
Subjective SES	13.45	1	<.001
Rural*Subjective SES	2.86	1	.09
Error		846	

*CABG* coronary artery bypass graft surgery, *SES* socioeconomic status.

Patients who had coronary artery bypass graft surgery also reported significantly lower CR barriers (1.90 ± 0.74 versus 2.12 ± 0.71, p < .001) compared to those who did not undergo this type of surgery. Given this was a novel finding, post-hoc comparisons of CR barriers among patients who had bypass surgery versus those who did not was performed. Participants who had bypass surgery rated the following barriers significantly lower than patients who had not: I didn’t know about CR (p < .001), I don’t need CR (p < .001), I find exercise tiring or painful (p < .001), time constraints (p < .001), I don’t have the energy (p < .001), My doctor didn’t feel it was necessary (p < .001), Many people with heart problems don’t go to CR and they are fine (p < .001), I can manage on my own (p < .001), and I prefer to take care of my health alone not in a group (p < .001).

## Discussion

Upon comprehensive assessment of CR barriers in this broad sample of cardiac outpatients, this study confirmed that rural inhabitants and patients of low SES experience significantly greater barriers to CR when compared to their more resourced counterparts. Analyses also demonstrated that bypass patients may experience fewer CR barriers than patients who have other indications for CR. To our knowledge, this is a novel finding.

One of the most important findings is the systemic barrier whereby patients of low SES were less likely to be referred to CR than their high SES counterparts. Given referral is a pre-requisite for enrollment, there should be a focus on interventions to improve referral systems so all patients, regardless of SES, have the same likelihood of being offered CR. Indeed, research by our group has demonstrated that systematic referral strategies result in significantly higher rates of CR referral and enrollment for patients of low SES, when compared to usual referral practices [34].

Given the burden of risk factors and poor outcomes demonstrated in rural cardiac patients and those of low SES in the literature [7], systematic identification and modification of barriers in these populations is warranted, to optimize their use of proven CR services. Interventions involving motivational communications delivered through letters, telephone calls, and home visits, as well as the use of liaison healthcare providers to support coordination of care, have all been shown to be effective in increasing uptake of CR [35,36]. Moreover, triage to structured and monitored home-based CR programs could enable rural inhabitants to overcome many of their identified barriers such as distance, and low SES patients to overcome many of their barriers such as transportation and costs (although this has not been tested). While these strategies have been known now for well over a decade, there has been a widespread failure to implement them. As pointed out by Valencia et al. in their recent review [7], when noting that home-based CR has not been widely implemented, this may be due to CR funding models. Similarly, it is likely that resource constraints are to blame for the lack of broad implementation of other known strategies as well.

Most recently, interventions tailored to overcoming identified barriers and improving CR utilization in these *under-served* populations are being empirically tested. For instance, recognizing that healthcare providers should identify under-served populations prior to discharge from the hospital, Meillier et al. [24] have developed a system to screen inpatients’ educational attainment as well as social support. They have tested the feasibility of their “social differentiation” approach, and go further to triage identified patients to an augmented model of CR. Their preliminary results were promising, suggesting high rates of program adherence in both models of care.

The novel finding that bypass surgery patients may experience significantly fewer barriers to CR is consistent with previous research which has shown greater CR utilization in bypass, when compared to percutaneous coronary intervention patients, for instance [37]. Upon reflection on the items which differentiated between the bypass and non-bypass patient barriers, the issue seems to center on lack of perceived need for CR. This appears to be the case for both patients and providers, although this contention warrants investigation prior to such interpretation. Indeed, from the patient’s perspective, those receiving bypass surgery likely do have more severe disease than those undergoing percutaneous coronary intervention. Moreover, there has been less research establishing the benefits of CR post-percutaneous coronary intervention when compared to bypass surgery, however CR is indicated in both instances. Motivational interviewing [25] could be helpful in addressing these non-logistical barriers identified by non-bypass patients, including perceptions that the norm is *not* to attend CR, and that they can manage their disease without the support of a CR program.

### Limitations

There are several limitations to this study. First, recall bias may be at play as a result of the amount of time that would have elapsed between healthcare provider interactions where CR may have been discussed, and completion of the one-year follow-up survey when the CRBS was administered. Second, patient-report of CR utilization may be inaccurate. However, previous research by our group has shown relatively high concordance between patient- and chart-report of CR utilization [38]. Moreover, patient-report of perceived healthcare provider and health system-level CR barriers may be biased. Third, generalizability of the findings are limited by some selection and retention biases in the sample, and to health care systems where CR services are not paid out-of-pocket by patients. Fourth, the indicator of rurality (i.e., drive time of greater than 30 minutes to acute care) may not accurately categorize patients. For instance, some CR programs are offered outside of acute care hospitals. Moreover, we did not observe a higher burden of risk factors in our rural compared to urban sample. In future studies, an alternate indicator such as distance to the closest CR site should be applied. Finally, due to the nature of the cross-sectional study design, causal conclusions cannot be drawn.

In conclusion, this study confirmed that rural inhabitants and patients of low SES experience greater barriers to CR compared to their more urban, high SES counterparts. The barriers more strongly-endorsed by rural patients and those of low SES appeared at the patient, provider and health system-levels. Indeed, as raised in the recent review paper by Valencia et al., [7] remedying these access disparities will accordingly require a multi-level approach. It is time for broader application of proven strategies to promote greater CR enrollment, and to develop and test tailored interventions to address the primary barriers identified for these vulnerable subpopulations of patients.

## Competing interests

The authors declare that they have no competing interests.

## Authors’ contributions

SS contributed to writing the paper, initiating the study, designing the study, and analyzing the data. PO contributed to writing the paper. RDR contributed to writing the paper. TM contributed to writing the paper. SLG (the guarantor) and contributed to writing the paper, initiating the study, and monitoring progress. All authors read and approved the final manuscript.
